# Shisha smoking in selected nightclubs in Nigeria

**DOI:** 10.11604/pamj.2019.33.136.17149

**Published:** 2019-06-24

**Authors:** Victor Olufolahan Lasebikan, Bolanle Adeyemi Ola, Tiwatayo Olufolahan Lasebikan

**Affiliations:** 1 Department of Psychiatry, College of Medicine, University of Ibadan, Ibadan, Nigeria; 2 Department of Behavioral Medicine, College of Medicine, Lagos State University, Ikeja, Nigeria; 3 Yaba Psychiatric Hospital, Lagos, Nigeria

**Keywords:** Shisha-smoking, nightclubs, cigarette-smoking, gender-difference, public-health

## Abstract

**Introduction:**

Shisha consumption is a growing public health issue all over the globe and public health awareness about its deleterious health consequences is still not sufficiently raised.

**Methods:**

In this location-based study of nightclubs in Ibadan, Nigeria, 633 patrons of selected nightclubs were interviewed in order to obtain information on prevalence, correlates and predictors of shisha smoking.

**Results:**

The overall prevalence of shisha smoking was 7.1%. The age of initiation into shisha smoking was lower among women, p = 0.03, but men were significantly more likely to be more frequent users, daily or weekly users, p < 0.001 and also to be current cigarette smokers, p = 0.03. There was no significant gender variability in the stage of readiness to quit. Regression analysis showed that after adjusting for age, the predictors of shisha smoking were: cigarette smoking, OR = 4.83, 95% CI (1.49-15.70) and more than 12 years of education, OR = 7.55, 95% CI (1.88 - 30.37), while being a rural dweller was a protective factor, OR = 0.05, 95% CI (0.01-0.20).

**Conclusion:**

Shisha smoking has emerged as a prevalent public health issue in Nigeria. There is a need for an immediate response from policy providers towards shisha smoking intervention in Nigeria.

## Introduction

Nicotine has been documented as a preventable cause of disease and death worldwide [1]. While its impact on high-income countries is decreasing, it continues to severely affect low and middle-income countries, including Nigeria. For example, annual mortality from tobacco use worldwide is estimated to be about 5 million, accounting for 1 in every 5 male deaths and 1 in 20 female deaths of those over the age of 30 [2]. It is projected that using current smoking patterns, annual tobacco deaths will rise to 10 million by 2030, most of them in low-income countries [2]. This trend highlights the urgent need to examine patterns of nicotine use in its different forms, particularly in Nigeria, one of the most populous countries in the world [3]. One form of tobacco smoking, referred to as shisha, hookah, hubble, bubble or narghile, has recently been gaining increased popularity in many developed and developing countries [4-7]. In Nigeria, shisha smoking has rapidly become increasingly popular in major cities. Factors mediating this sudden trend are variable, including smoking for pleasure, smoking for its stimulating effect, experimentation, or perceived safety compared with cigarette smoking [8]. The vogue is rapidly pervading society and is commonly practiced by university undergraduates, adolescents and the older population in restaurants and hotels and at social gatherings [8]. Several authors have suggested many reasons for its increasing acceptance and appeal, including the perception of its less harmful effects compared to cigarette smoking [9, 10], the perception of it being less hazardous to health because of the addition of fruit flavours [9], the perception of it being less addictive than cigarette smoking [11] and its societal relevance as a symbol of fashion [11]. However, the smoke from shisha contains 6.5 times more carbon monoxide, 1.7 times more nicotine, 46 times more tar and dangerous heavy metals (Djordjevic *et al* 2000). Thus, shisha smoking puts users at high risk of addiction, cardiovascular diseases, respiratory diseases and infections such as tuberculosis and herpes due to sharing shisha mouth pieces [12].

While the World Health Organization (WHO) has initiated measures to combat tobacco smoking [13], less attention has been given to understanding the different forms and patterns of smoking, which could be important in guiding strategies against the use of tobacco, in this instance shisha, a growing tobacco product in Nigeria. There is a need to examine the prevalence of use and possible correlates in order to guide policies and strategies to address this emerging problem. Shisha (hookah, narghile, “water-pipe”) smoking is now regarded in public health as a global tobacco epidemic. This is because epidemiological data from countries, where surveillance of shisha is prioritized have shown alarming trends. While in the Middle East, shisha has replaced cigarettes as the most popular method of tobacco use among youth, it is becoming second to cigarettes in other parts of the world [14]. There is ample evidence that shisha smoking is harmful and addictive, and that it can provide a gateway to cigarettes, as well as thwart cigarette smoking cessation efforts [15]. The emergence of shisha smoking around the world has prompted research into its toxic content. Compounds commonly found in shisha smoke include carbonylic compounds, CO, tobacco-specific nitrosamines, primary aromatic amines, furanic compounds, polycyclic aromatic hydrocarbons, heavy metals, and volatile organic compounds, with most yields being higher for a shisha smoking session than for a single cigarette session [16].

Specifically, shisha smoking has been documented to have hypertensive effect [17], it is also a risk factor for respiratory diseases such as chronic obstructive airway disease [18], lung, oesophageal and gastric cancers [19], obstetric and perinatal complications such as birth defects [20] and infectious diseases due to viral isolates such as herpes from the waterpipe [21]. Unfortunately, developing, monitoring, intervention and regulatory/policy frameworks specific to shisha smoking is not a public health priority in Nigeria. In Nigeria, despite an upsurge of discotheques and nightclubs where people drink and smoke, there is a very low level of awareness about the harmful effects of shisha. Since night clubbing is very common not only among adolescents and young adults, but also across other age groups in Nigeria [22], we conceived that such places would be suitable potential catchment sites of shisha smoking that may be representative of such activities in Nigeria. Therefore, in this study, our primary aims were to determine the prevalence and sociodemographic correlates of shisha smoking, as well as to analyze factors associated with gender differences in shisha smoking among a representative sample of nightclub patrons in Ibadan, Nigeria. We used a location-based sampling method, because we believed it would be appropriate for obtaining answers to our research questions from people within the contexts of nightclubs who might otherwise be missed in other research settings. In this study, we defined a “nightclub” as a place that is open from the evening until early morning, having facilities such as a bar and disco or other entertainment.

## Methods

**Study setting and background information on the area:** the study area was Ibadan, the capital of Oyo state, Nigeria. The city is located in the southwestern part of the country. It had a population of about 2.5 million people as of 2009. Ibadan is divided into eleven local government areas (National Population Commission, 2010). The study took place between June 3 and August 2, 2015.

**Sampling technique and procedure:** a systematic random sampling technique was employed for sample selection. Ibadan is comprised of 11 local government areas (LGAs). The 11 LGAs were listed and divided into wards, a ward being a subunit in the LGA as designated during the national census in Nigeria (National Population Commission, 2010), after which, two wards were randomly selected from each local government. The wards were further divided into enumeration areas [23]. One enumeration area was randomly selected in each of the wards selected and a social club was randomly selected in each enumeration area, thereby making a total of 22 social clubs spanning the 11 local governments in the state. The nightclubs were thereafter classified into three groups, urban, semi-urban and rural, based on a local government funding allocation classification. Two nightclubs were then randomly selected from each of these three groups.

**Sample size:** the minimum sample size was obtained using sample size estimation for a descriptive cross-sectional study

$$\text{n}={\text{Z}}^{2}\text{pq}/{\text{d}}^{2}$$

where Z = 1.96, p = anticipated prevalence of shisha smoking (50%) in the absence of previous data, q = 1-p = (50%), d = 0.05 (precision at 95% CI) [24], thus the minimum sample size was 384. However, considering the usual low response rate known to addiction researchers, we interviewed all consenting adults encountered at the nightclubs as soon as they opened at 6pm.

**Sampling process:** In this study, all consenting subjects (n = 740) were recruited using a non-probability purposive sampling. The first participant was randomly selected and subsequent ones consecutively selected until all participants for the day had been interviewed at each of the study sites. Data collection was finalized when it was believed that all new participants had been recruited during the proposed data collection period and no new participants were recruited afterwards (data saturation). All participants were allocated code numbers for the purpose of identification. The response rate was 90%.

### Measures

**Sociodemographic characteristics:** in the face-to-face interview, we obtained sociodemographic data from all participants using a sociodemographic questionnaire that yielded information on characteristics such as age, gender, marital status, occupation, education, income, age of initiation, reason for use and concurrent smoking.

**Prevalence of shisha use:** the Alcohol, Smoking and Substance Involvement Screening Test (ASSIST) was used to obtain the prevalence of shisha use in the past three months. This instrument was developed by the World Health Organization for screening for drugs and alcohol in high-prevalence settings [25]. We obtained the current prevalence of shisha use from Q2: “in the past 3 months, how often have you used shisha?” The first question in the instrument was “in the past 3 months, how often have you used any tobacco product?” We also obtained information about cigarette smoking from this question. Responses were: “not at all”, “once or twice”, “monthly”, “weekly” and “daily/almost daily@. The coding of the responses was used to generate a score, which was then used to determine the type of brief intervention to follow. ASSIST scoring: for tobacco or any drug, 0-3 (low risk), 4-26 (moderate risk), 27+ (high risk).

**Other data:** we also developed a profoma where information on the reasons for use and stage of readiness to quit were recorded.

**Reasons for use:** the question on the reasons for shisha smoking was open-ended and responses were classified as: “safer than cigarettes”, “social class” and “flavour”. Other reasons such as “I just started it”, “I have no reasons for this”, “it is part of life”, “one of those things” were classified under a common heading “no specific reason, it is the current trend”.

**Readiness to quit:** the five stages of readiness to quit were: pre-contemplation, contemplation, preparation, action and maintenance in accordance with Prochaska and DiClemente [26]. The questions asked were as follows: Pre-contemplation: do you have any intention to change shisha smoking in the foreseeable future? Response was either “yes” or “no”. Contemplation: are you aware that your shisha smoking is a problem and are you seriously thinking about overcoming it, but have not yet made a commitment to take action? Response was either yes" or no". Preparation: are you intending to stop shisha smoking in the next month? The response was either “yes” or “no”. Action: have you successfully achieved abstinence for a period of from one day to six months in the past year? The response was either “yes” or “no”. Maintenance: have you prevented a relapse to consolidate the gains attained during shisha abstinence? The response was either “yes” or “no”.

**Data collection:** all interviewers had previously been involved in addiction research. They also received two weeks of training consisting of an initial three-day training session by the authors, followed by a further two days of debriefing and reviews, after each interviewer had conducted two pilot interviews in the field. A supervisor was responsible for verifying the completeness of every questionnaire. Field checks were made to ensure the correct implementation of the protocol and full adherence to the interview format.

**Ethics and consent to participate:** ethical approval was obtained from the Ethical Review Committee of the Department of Planning, Research and Statistics, Ministry of Health, Oyo state, Nigeria in accordance with the Declaration of Helsinki to ensure adherence to proper ethical standard and informed consent was obtained from each participant in the study. Permission was also obtained from each of the open-joint operators. Participants were duly informed of the objectives of the study and anonymity was maintained as serial numbers and not names were used during the entire study period, from period of data collection to include data entry and analysis.

**Analysis:** the data generated were entered into the system and then analysed using the Statistical Package for the Social Sciences (SPSS) version 20.0 software. Associations between the prevalence of shisha smoking and demographic characteristics were calculated using Chi square statistics. Gender differences in the characteristics of shisha smokers were analysed using Chi square statistics. Binary regression analysis was used to determine the effects of multiple confounding variables on the outcome variable, namely the prevalence of shisha smoking. We adjusted for age, but not for gender, because gender was not significantly associated with shisha smoking. The dependent variable was shisha smoking, while the independent variables were educational, employment and marital status, area of residence, smoking, and income. Income was excluded from the final output because of collinearity with employment status. All tests of significance were set at 95% confidence interval, p < 0.05.

## Results

**Characteristics of the sample** in the current study, we set out to interview all consenting participants in the various study sites. They totalled 740 in all; however, data were complete for 633 respondents. The mean age (SD) of all shisha users was 45.98 (11.41) years and for non-users 39.13 (12.27) years, which was significant, t = 3.6, p < 0.001. The prevalence of shisha smokers was 45 (7.1%). A significantly higher proportion of shisha smokers also had more years of education, X^2^ = 40.1, p < 0.001, were high-level professionals, X^2^ = 20.4, p < 0.001, were married, X^2^ = 4.6, p = 0.03, lived in urban areas, X^2^ = 54.6, had a higher income, t = 15.8, p < 0.001 and were simultaneously current cigarette smokers, X^2^ = 6.7, p = 0.01 (Table 1). The age of initiation into shisha smoking was significantly lower in women, t = -2.9, p = 0.006. Despite this, the frequency of use was significantly higher in men, X^2^ = 14.3, p < 0.001, but there was no significant gender variability in the stage of readiness to quit, p = 1.0. A significantly higher proportion of the men were also current cigarette smokers, X^2^ = 4.5, p = 0.03, and there was a significant gender difference in the reason for its use, with the highest proportion of women smoking shisha because of its flavour and men having no specific reason and stating that it was the trend, X^2^ = 15.6, p < 0.001 (Table 2). After adjusting for age, regression analysis showed that predictors of shisha smoking were: cigarette smoking, OR = 4.83, 95% CI (1.49 - 15.70) and more than 12 years of education, OR = 7.55, 95% CI (1.88 - 30.37), while being a rural dweller was a protective factor, OR = 0.05, 95% CI (0.01 - 0.20) (Table 3).

**Table 1:** Sociodemographic characteristics of shisha smokers

	User (45)		Non-user (628)		Statistics	P
	N	%	N	%		
Age (years)						
< 35	6	2.5	235	97.5	14.0, df(4) X	0.007
35-44	14	8.3	155	91.7		
45-54	16	11.7	121	88.3		
55-64	7	10.3	61	89.7		
>64	2	11.2	16	88.7		
Mean age (SD)	45.98	(11.41)	39.13	(12.27)	3.6t	< 0.001
Education						
None	4	3.0	129	97.0	40.1, df(3) X	< 0.001
Elementary	19	6.1	293	93.9		
Secondary	10	6.5	143	93.5		
Post-secondary	12	34.3	23	65.7		
Gender						
Male	36	7.2	461	92.8	0.06 X	0.8
Female	9	6.6	127	93.4		
Employment						
Unemployed	2	2.3	85	97.7	20.4, df(4)X2	< 0.001
Unskilled	2	5.4	35	94.6		
Semi-skilled	3	2.4	121	97.6		
Skilled	15	6.6	211	93.4		
High-level Professional	23	14.5	136	85.5		
Marital status						
Married	34	8.9	349	91.1	4.6 X	0.03
Unmarried	11	4.4	239	95.6		
Residence						
Urban	29	19.5	120	80.5	54.6, df(2) X	< 0.001
Semi-urban	13	8.7	136	91.3		
Rural	3	0.9	332	99.1		
Income in US dollars						
Mean (SD)	2.153.33	(776.07)	598.02	324.79	-15.8t	< 0.001
Cigarette smoking						
Yes	41	8.6	433	91.4	6.7	0.01
No	4	2.5	154	97.5		

X: chi square; t: t test;

**Table 2:** Characteristics of shisha smoking by gender in the past three months

	Male		Female			
	N = 32	%	N = 13	%	Statistics	P
Mean age at initiation (SD)	40.06	(10.85)	30.15	(8.7)	2.9 t	0.006
Frequency of use						
Daily	29	84.6	13	43.3	14.3 X	< 0.001
Weekly	1	9.0	-	-		
Monthly	2	5.1	5	16.7		
Once or Twice	-	1.3	12	40.0		
Ready to quit						
Pre-contemplation	25	71.4	10	28.6	0.03 X	1.0
Contemplation	5	71.4	2	28.6		
Preparation	2	66.7	1	33.3		
Cigarette smoking						
Yes	31	75.6	10	24.4	4.5 X	0.03
No	1	25.0	3	75.0		
Reason for use						
No Specific Reason (Trend)	21	91.3	2	8.7	15.6 X	< 0.001
Flavour	3	27.3	8	72.7		
Social Class	3	60.0	2	40.0		
Safer than Cigarette	5	83.3	1	16.7		

t = t test; X: Chi square

**Table 3:** Odds of shisha smoking

Variables	OR		95% C.I.	
		Lower	Upper	P
**Age (years)**				
< 35	1			
35-44	2.36	0.68	8.23	0.3
45-54	2.61	0.70	9.83	0.2
55-64	4.92	1.20	20.25	0.03
>64	2.62	0.38	17.92	0.3
**Cigarette**				
No	1			
Yes	4.83	1.49	15.70	0.01
**Employment**				
Unemployed	1			
Unskilled	0.50	0.05	4.83	0.5
Semi-skilled	0.64	0.08	4.91	0.6
Skilled	1.11	0.20	6.17	0.9
High-level Professionals	2.31	0.40	13.23	0.3
**Marital status**				
Married	1			
Unmarried	1.04	0.40	2.66	0.9
**Residence**				.
Urban	1			
Semi-Urban	0.70	0.31	1.60	0.4
Rural	0.05	0.01	0.20	< 0.0001
**Years of education**				
Nil	1			
1-6	1.68	0.48	5.85	0.4
7-12	0.97	0.27	3.50	1.0
> 12	7.55	1.88	30.37	< 0.01

## Discussion

This study identified distribution patterns, correlates and knowledge that could guide shisha - specific prevention and intervention strategies to curb its spread in Nigeria. To the best of our knowledge, this is the first published reports on the subject in Nigeria, the most populous black nation in the world. Importantly, this study addresses one of the critical research needs identified by the WHO, namely the pattern of shisha smoking in particular countries and cultures. Research on shisha use in Africa is limited and the little research which has been conducted was in South Africa among university students [27] and in a community survey in Ethiopia [28]. Far more studies have been reported in North African countries, including Egypt [29, 30] and Tunisia [31]. While the use of shisha fits a common global pattern of students embracing shisha smoking as a social experience, this study specifically explores the use of shisha in nightclubs in Nigeria, in order to include a variety of age groups and socioeconomic classes.

**Main findings:** the results of the study show that the prevalence of shisha smoking in our sample was 7.1% and that shisha smokers were significantly older, with more years of education, more likely to be skilled workers and high - level professionals, more likely to be married and live in urban areas and also more likely to be high-income earners. There was no significant gender difference in the prevalence of shisha smoking and none of the shisha users were ready to quit. We found that the current prevalence of shisha in this study is higher than the prevalence of tobacco products other than cigarette smoking (0.8%) in the entire population over 15 years [32]. This pattern is in line with anecdotal evidence of shisha use proliferation in other countries in regions such as South Africa, Ethiopia, Egypt and Tunisia [27-31]. We found an overall prevalence of shisha smoking of 7.1%, with 7.2% in men and 6.6% in women. Except for Vietnam, the figure in men is higher than that reported for any other country in a cross-country comparison of water pipe use involving 13 low and middle-income countries from the Global Adult Tobacco Survey (GATS) between 2008 and 2010 involving Bangladesh, Brazil, China, Egypt, India, Mexico, the Philippines, Russia, Thailand, Turkey, Ukraine, Uruguay and Vietnam [33]. The prevalence found among women in the current study is also higher than figures reported among women in Russia, Ukraine, Turkey, India, Egypt, Bangladesh, Vietnam, Uruguay, Brazil, China and Thailand, with no figure available for women in the Philippines and Mexico [33]. Nevertheless, higher figures, namely 18.9 % in males and 23.1 % in females have been reported among adult populations, in Jordan [34]. In contrast to tobacco use [35, 36], we found a high prevalence of shisha use among higher socioeconomic classes and those with at least a high school education. A similar observation was made among men in Russia and Ukraine in the 2008-2010 GATS [33]. It may be conceptualized that high income or being a member of a well-educated group embraces shisha as symbolic of the social class experience. It is possible that they consider shisha smoking prestigious and an indication of having a modern standard of living. This corroborates a recent report on a cross section of individuals interviewed by a journalist in the capital city of Nigeria [8]. Further studies are needed to explore the influence of social class on shisha smoking. With the high proportion of users in this group, they are more likely exposed to the inhalation of toxic materials in shisha. In other words, the harmful consequences of shisha smoking might be prevalent among this high income sector of Nigerian society. Shisha use can be characterized as a growing and unrecognized public health issue among the productive sector of the population and there is a need to raise public policy awareness about this problem.

**Why these high rates?** the seemingly high rates of shisha smoking in the current study compared to other studies could be attributed to the study population and the method of data collection. The population studied were nightclub patrons, in a location which is the predominant catchment area for shisha smokers [37], rather than a general population survey as in the GATS [33]. We also found that the prevalence of use was higher among the urban and semi-urban dwellers than those in rural areas, a reversal of the distribution of cigarette smoking in Nigeria and other countries of the world [35, 36]. Our observation of a higher prevalence in the urban setting has been similarly reported in Russia, Turkey and Ukraine during the 2008-2010 GATS, although rural men had a higher prevalence than their urban counterparts in Vietnam and Egypt [33].

**Gender variations:** we found that men were significantly more frequent Shisha smokers than women, despite the lack of significant gender differences in the overall prevalence of shisha use in the past three months. There are indications of an alarmingly high rate of shisha use among both men and women in some Arabic countries [34] and frequent shisha smoking is found in nightclubs, where it is often consumed along with alcohol [37]. Nevertheless, despite the frequent use of shisha among these nightclubs patrons, there was a general lack of readiness to quit using it, irrespective of gender. This finding is similar to reports of studies carried out in some other parts of the world [9, 10, 38]. It is possible that the general misperception that shisha is not hazardous could be responsible for this finding. Shisha smokers usually believe that it is less harmful than cigarettes because the smoke is filtered through water [5]. This misconception may be reinforced by marketing tools and social pressures. Unlike cigarette packaging, which carries mandatory health warnings, shisha products are commonly sold with no such health warning. Labels on shisha packs claim their product is “natural” or “free of chemicals” and powered by inviting varieties of flavours, suggesting there is a risk for continued use, and exploration by non-users. This has huge public health implications and carries an additional risk for women with regard to their reproductive health [39] and is particularly pertinent considering our finding that significantly more women compared with men reported “flavour” as their commonest reason for shisha use and also that a significantly higher proportion of women had younger age of initiation into use than men.

**Why this demographic reversal?** our observation of a higher prevalence of shisha smoking in urban dwellers, older men and women, and the lack of gender difference in the shisha smoking rate appears to be a paradox to the well-known demographic correlates of cigarette smoking. The question is, what exactly could be responsible for this reversal? It may suggest a different dynamics in the aetiology of shisha smoking. Indeed, there are reports that the resurgence in the popularity of shisha smoking after an initial decline in the 20^th^century coincided with the manufacturing of sweetened tobacco known as “maassel”, specifically made for water pipe smoking [9]. The aggressive marketing campaigns via the media and a myriad of flavours has contributed tremendously to the massive increase in the popularity of shisha [40]. The uncommon demographic trend in shisha smoking may be partially ascribed to its perception as being less harmful than cigarettes, coupled with its flavours, thereby making it a substitute for cigarettes among older men and women in urban areas.

**Reasons for shisha smoking:** the reasons for smoking shisha reported in this study provide important information about the habit as well as an explanation for the current situation of shisha smoking in Nigeria. Firstly, the top two most common reasons for smoking shisha were: “no specific reason, shisha is trendy”, implying shisha is gaining popularity among nightclub patrons and flavour. It appears that shisha smoking has become a contextual norm in nightclubs in Nigeria. The trend cuts across adolescents and the older population in restaurants and hotels and at social gatherings [8]. Our study replicated previous findings [10, 41] which found that shisha smokers were also more likely to be cigarette smokers, most especially men. This highlights, the need for more public health initiatives regarding tobacco product use in any of its forms in Nigeria. Despite the high prevalence of shisha smoking in our sample, the vast majority of users were not ready to quit. This may be due to its misperception as being healthier than cigarette smoking or because of its highly addictive nature. For example, the exotic flavour and the attractive architecture of the nicotine delivery device whereby water vapour acts instead of smoke, creates a misleading and false perception about its safety [4]. More toxic chemicals than produced by cigarettes are delivered per session of shisha, which consumes approximately 10-20 g of tobacco and 5 g of charcoal [42]. Compared to cigarette smoking, 6.5 times more carbon monoxide, 1.7 times more nicotine and 46 times more tar, along with other lung carcinogens and heavy metals, are delivered during shisha smoking [12]. Thus, shisha is more addictive and more carcinogenic than cigarette smoking.

**Research and policy implications:** our study locations were in nightclubs, which highlighted, in agreement with other studies, the acceptability of misperceptions of harmlessness or less harmfulness than cigarettes and lack of regulations governing shisha smoking [43, 44]. A major factor in the spread of the “shisha epidemic” is that tobacco control policy in many parts of the world generally does not extend to hookahs [14]. The picture appears even gloomier in Nigeria, a country where not even a tobacco control bill for cigarettes has yet been implemented. Our finding calls for policy driven health campaigns and educational awareness programs, that will increase public knowledge regarding the hazardous and harmful consequences of shisha use. It is also necessary to develop evidence-based, counter-advertising programs to create understanding of its hazards. These measures have the potentials to be effective in Nigeria, given that shisha smoking is linked with higher levels of education. Our findings also have serious implications for shisha smoking cessation, especially since none of the users were ready to quit.

**Strength and weaknesses:** the major strength of our study is the large sample size and the study sites, with our locations being drawn from a representative sample of the larger nightclub community. To deal with the shisha problem, shisha - specific models and measures are needed to address the associated risk factors that this study has identified. We are mindful of the limitation of obtaining substance use information due to the sensitive nature of the subject. This was, however, not an issue by virtue of the “pride” associated with shisha use among our study participants. Nevertheless, some of the data obtained could be masked by under-reporting as influenced by the location-based sampling technique utilized, which is highly vulnerable to selection bias. However, we adopted a statistical method that was appropriate for the nature of this study as it was the only feasible method for a limited number of primary data source. This method also had the advantage of exploring contextual substance use within the study setting.

## Conclusion

In Nigeria, shisha smoking is prevalent in nightclubs and has peculiar demographic correlates different from cigarette smoking. Although men were more likely to be regular users, the early age at initiation into use in women is an important epidemiological finding, especially regarding their reproductive health. The high prevalence of shisha smoking in our sample is also a major public health issue, given our findings that none of the shisha smokers were ready to quit and also considering that the majority of the users were high level professionals in their productive stage of life. Our findings call for an immediate public health response from policy providers towards shisha smoking intervention in Nigeria.

### What is known about this topic

- Shisha smoking is gaining increasing acceptance and appeal all over the world;
- Shisha smoking is generally perceived as less harmful than cigarette smoking.

### What this study adds

- Shisha smoking is more prevalent among older men and women;
- Unlike cigarette smoking, there is no gender difference in the rate of shisha smoking.

## Competing interests

The authors declare no competing interests.
